# Clinical Characteristics and Degree of Glycemic and Cardiovascular Risk Factor Control in Patients with Type 1 Diabetes in Catalonia (Spain)

**DOI:** 10.3390/jcm10071536

**Published:** 2021-04-06

**Authors:** Gabriel Gimenez-Perez, Josep Franch-Nadal, Emilio Ortega, Manel Mata-Cases, Albert Goday, Jordi Real, Angel Rodriguez, Bogdan Vlacho, Dídac Mauricio

**Affiliations:** 1 Endocrinology Unit, Department of Medicine, Hospital General de Granollers, 08402 Granollers, Spain; ggimenez@fphag.org; 2 DAP-Cat Group, Unitat de Suport a la Recerca Barcelona, Fundació Institut Universitari per a la Recerca a l’Atenció Primària de Salut Jordi Gol i Gurina (IDIAPJGol), 08007 Barcelona, Spain; josep.franch@gmail.com (J.F.-N.); manelmatacases@gmail.com (M.M.-C.); jordi.real@gmail.com (J.R.); bvlacho@idiapjgol.org (B.V.); 3 CIBER of Diabetes and Associated Metabolic Diseases (CIBERDEM), Instituto de Salud Carlos III (ISCIII), 28029 Barcelona, Spain; 4 Primary Health Care Centre Raval Sud, Gerència d’Atenció Primària Barcelona Ciutat, Institut Català de la Salut, 08001 Barcelona, Spain; 5 Department of Endocrinology & Nutrition, Institut d’Investigacions Biomèdiques August Pi Suñer, CIBEROBN, Hospital Clínic Barcelona, 08036 Barcelona, Spain; eortega1@clinic.cat; 6 Primary Health Care Centre La Mina, Gerència d’Atenció Primària Barcelona Ciutat, Institut Català de la Salut, 08930 Sant Adrià del Besós, Spain; 7 Department of Endocrinology & Nutrition, Hospital del Mar, 08003 Barcelona, Spain; 88565@parcdesalutmar.cat; 8 Department of Medicine, Universitat Autònoma de Barcelona, CIBEROBN, 08193 Barcelona, Spain; 9 Department of Clinical Research, Lilly, S.A., 28108 Madrid, Spain; rodriguez_angel@lilly.com; 10 Department of Endocrinology & Nutrition, Hospital de la Santa Creu i Sant Pau, 08041 Barcelona, Spain; 11 Faculty of Medicine, University of Vic-Central University of Catalonia, 08500 Vic, Spain

**Keywords:** type-1 diabetes, cardiovascular risk factors, glucose control, complications

## Abstract

Background: This study aims to evaluate the clinical characteristics, complications, degree of glycemic control, and cardiovascular risk factor control in patients with type 1 diabetes in Catalonia (Northwest of Spain). Methods: Cross-sectional study using a database including clinical, laboratory, and treatment data. Patients with an ICD10 diagnosis of type 1 diabetes were included, excluding those treated with glucose-lowering agents other than insulin, or treated only with basal insulin two years after diagnosis. Results: 15,008 patients were analysed. Median IQR age was 42 (31–53) years, diabetes duration 11.8 (6.8–16.0) years, 56.5% men. Median (IQR) HbA1c was 7.9% (7.1–8.8). Microvascular complications were present in 24.4% of patients, 43.6% in those with a diabetes duration >19 years. In presence of known cardiovascular disease 69.3% of patients showed an LDL-C concentration >70 mg/dL, 37% had a systolic blood pressure >135 mmHg and 22.4% were smokers. Conclusions: This study provides a reliable snapshot about the clinical situation of a large population of patients with T1D in Catalonia, which is similar to that of other western areas. The lack of adequate control of cardiovascular risk factors in a significant proportion of patients with cardiovascular disease deserves a more detailed analysis and urges the need for improvement strategies.

## 1. Introduction

Type 1 diabetes (T1D) is one of the most prevalent chronic diseases during childhood and youth. The estimated prevalence in Spain is around 15 cases per 1000 in people under 20 years of age [1], with an incidence rate of 11.8 per 100,000 per year in people up to 30 years [2,3], rendering a prevalence of 3 per 1000 in the adult working population [4]. Contrary to some other countries, there is no evidence of an increase in the incidence over time. However, the current figures are similar to those of the countries considered to have the highest incidences [2,5] of T1D.

Although T1D accounts for a minor proportion of patients with diabetes globally, it is still a major health issue because it often affects young people who will live with the disease for many years, increasing the probabilities of microvascular and macrovascular complications [6,7]. As a consequence of these complications, individuals with T1D have a reduced life expectancy [8]. Further, their health-related quality of life, their work productivity, and their daily physical activities are adversely affected [9]. An adequate, goal-directed treatment of hyperglycemia can reduce the risk of microvascular and macrovascular complications, including cardiovascular mortality in patients with T1D [10,11]. In addition to the treatment of hyperglycemia, it is highly plausible that the treatment of high cholesterol and blood pressure has a role in the reduction of cardiovascular morbidity and mortality in T1D [12]. However, and despite significant improvements in cardiovascular morbidity and mortality [8], patients with T1D are still exposed to significant excess mortality compared to people without diabetes [13]. A better understanding of the clinical status and characteristics of individuals with T1D can help healthcare providers to develop strategies to improve patient care and to better adapt the management and treatment of the disease to internationally recognized standards of clinical practice [14]. With this in mind, this study sought to describe the clinical characteristics, burden of complications, degree of glycemic control, and cardiovascular risk factor control, as well as treatment in a large population-based cohort of patients with T1D in Spain.

## 2. Materials and Methods

We conducted a cross-sectional study including all patients with T1D registered with the Catalan Health Institute (CHI) of Catalonia, a Mediterranean region in north-eastern Spain. The CHI is a publicly owned health care organization providing healthcare to approximately 5.8 million citizens (80% of the region’s population). The rest of the Catalan population is served by other publicly funded non-profit organizations that in 2016 were not using the same electronic clinical station. CHI operates 280 primary health care centres with >3500 family physicians (FP). Every citizen is registered with a single FP. All FPs use the same clinical software called ECAP to record clinical information of their assigned patients. Prescribed medication is dispensed in private pharmacies, and once dispensed, it is recorded in a general database (CatSalut database). Typically, patients with T1D are attended at the specialist care setting bound to a hospital facility. However, prescriptions of chronic treatments and glucose control materials are provided at the primary care centers, guaranteeing that regardless of where clinical care is provided, the coverage of patients with T1D of any age by the ECAP and CatSalut databases is almost universal. Public hospital care in Catalonia is shared between CHI-owned hospitals and hospitals owned by non-profit organizations funded through agreements with the Catalan health authority (CatSalut).

In this study, data were retrieved from the SIDIAP database (https://www.sidiap.org/; accessed on 29 March 2021). SIDIAP is a computerized database containing anonymized patient records for people recorded with an FP in the CHI. SIDIAP includes data from ECAP (demographics, diagnoses, clinical variables, prescriptions, and referrals), laboratory test results directly obtained from ICH laboratories or manually entered by FPs in the ECAP (for data from laboratories not linked to ICH), medications dispensed in pharmacy offices (provided by the CatSalut database) and data of hospital discharges obtained from the Minimum Basic Data Set for Acute Hospitals provided through the CatSalut database. The SIDIAP database has been used previously to carry out several observational studies that evaluate different aspects of the natural history and treatment of type 2 diabetes in Catalonia [15,16].

Initially, all patients of any age with a diagnosis of T1D (International Classification of Diseases 10 [ICD-10] code E10) prior to 1 January 2017 were retrieved. To refine the patient selection, we applied restrictive criteria excluding patients with an E10 diagnosis treated with glucose-lowering agents other than insulin and those who were not treated with short acting insulins more than two years after the recorded date of diagnosis. The restrictive criteria were based on records of medications dispensed at pharmacies during the data collection period from 1 January 2016 through 31 December 2016, thus limiting the cross-sectional cohort to clients who had contact with the CHI system during this period. If not otherwise stated, the last recorded measure during the year 2016 was recorded. Data collected for the present analysis included age, sex, body mass index, duration of diabetes (2016 minus year of diabetes diagnosis), recorded cardiovascular risk factors (hypertension, dyslipidemia, smoking and obesity; last recorded entry, possibly before 1 January 2016), HbA1c, blood pressure (mean of the last three measures during 2016, if available), blood lipids, estimated glomerular filtration rate (GFR) using the CKD-EPI (Chronic Kidney Disease Epidemiology Collaboration) formula, urinary albumin/creatinine ratio, diabetes therapy (insulin type, adjuvant glucose-lowering medications). We also collected recorded the diagnosis of T1D complications (last recorded entry, possibly before 1 January 2016), i.e., diabetic retinopathy (ICD-10 codes E10.3, H36.0), diabetic nephropathy (ICD-10 code E-12) and related treatments (dialysis or renal transplant), diabetic neuropathy (ICD-10 codes E10.4, G63.2), peripheral artery disease (ICD-10 codes E10.5, I70.2, I73.8, I73.9, I79.2) and related comorbidities i.e., coronary heart disease (ICD-10 codes I20–25), cerebral vascular disease (ICD-10 codes I63, I64, I67.2-4, I67.8-9, I69, G45-46), heart failure (ICD-10 codes I150, I11.0, I13.0, I13.2) and cardiovascular procedures (coronary revascularization and lower extremity vascularization or non-traumatic amputation). We also collected data about acute diabetes complications, specifically recorded hypoglycemia (ICD-10 codes E10.0, E16.0, E16.2) (Table S1 in Supplementary Materials).

We analyzed the degree of control of cardiovascular risk factors according to the variables used in the Steno T1 risk engine [17,18] with slight modifications of the upper limits of normal values.

The study was approved by the Ethics Committee of the Primary Health Care University Research Institute Jordi Gol.

### Statistical Analysis

Medians and interquartile ranges (IQRs) or proportions were calculated for all variables (clinical characteristics, diabetes-related complications, and treatment). Descriptive analyses were conducted for the overall population, as well as stratified by sex, age group (<15, 15–40, and >40 years), diabetes duration (<10, 10–19, >19 years) and the absence/presence of cardiovascular disease where appropriated. Data management was performed using the R 3,6,0 software (https://www.r-project.org/; accessed on 29 March 2021).

## 3. Results

Initially, 23,591 patients with a diagnosis of T1D were identified of which 8583 were excluded after the application of the restrictive criteria, resulting in a final sample of 15,008 valid cases (Figure S1 in Supplementary Materials).

Clinical and laboratory characteristics are summarized in Table 1. The median age was 42.0 years and 56.5% were male. The median diabetes duration was 11.8 years and the median age at diagnosis was 27.9 years (Figure S2 in Supplementary Materials). The most common cardiovascular risk factors recorded were being overweight or obese (51.9%) followed by similar percentages of active smoking (26.1%), hypertension (23.0%), and hypercholesterolemia (22.8). Missing values for numerical variables are summarized in Table S2 (in Supplementary Materials).

Most of the variables did not show clinically significant differences between genders with the exception of a higher percentage of smoking (present or previous) in males, a lower median systolic blood pressure and higher median HDL-cholesterol in females, and globally fewer drug treatments also in females.

### 3.1. Glucose Control and Diabetes-Related Complications

**Figure 1:**
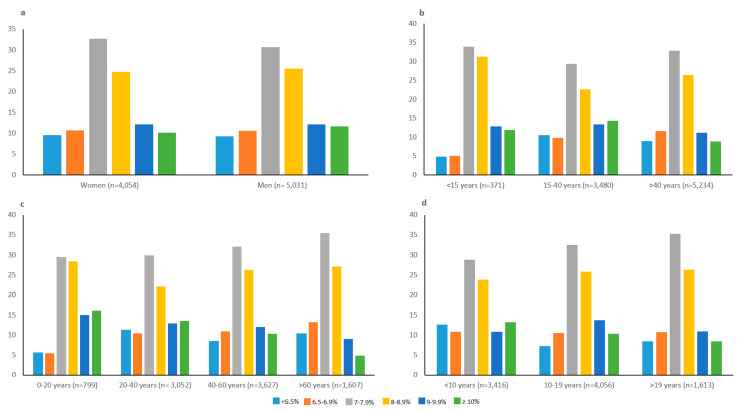
Percentage distribution across HbA1c categories according to sex (**a**), current age (**b**,**c**) and diabetes duration (**d**). All comparisons between groups were significant (*p* < 0.001).

For the entire population, median (IQR) HbA1c was 7.9% (7.1–8.8). Twenty percent of patients showed an HbA1c <7% and 21.3% an HbA1c ≥9%. Figure 1 depicts the percentage distributions across HbA1c categories according to sex (Figure 1a), current age (Figure 1b,c), and diabetes duration (Figure 1d).

There were no clinically meaningful differences in glucose control according to gender (Figure 1a).

The median HbA1c (IQR) levels were 8.1% (7.5–8.9), 8.0% (7.1–9.1), and 7.8% (7.1–8.7) for patients <15, 15–40, and >40 years of age, respectively. Slight differences were observed in the distribution across HbA1c categories according to current age with a tendency to better control with increasing age (Figure 1b). Thus, the percentage of patients with HbA1c <7% was 10% in young patients (<15 years of age) and 20.6% in older adults (>40 years of age). The corresponding percentages of patients with HbA1c ≥9% were 24.8% and 20%. However, it should be noted that the percentage of patients <15 years of age with recorded HbA1c levels was only 33.2% (371 patients). The same trend can be observed if patients are grouped according to 20-year (Figure 1c) or 10-year age ranges (data not shown).

The median HbA1c (IQR) levels were 7.9% (7.0–8.9), 7.9% (7.2–8.8), and 7.8% (7.1–8.7) for patients with a diabetes duration of <10, 10–19, and >19 years, respectively. There were no clear differences in glucose control based on the duration of diabetes (Figure 1d) except for the highest percentages of patients with less than 10 years duration at both ends of the HbA1c categories. However, the differences were small (maximum of five percentage points).

The prevalence of recorded hypoglycemia was 8.4%, with higher prevalence with increasing age and diabetes duration (9.7% in patients >40 years of age and 11.9% for diabetes duration >20 years).

Prevalence of chronic complications is shown in Table 2. Retinopathy was the most prevalent recorded complication. As shown, most complications were slightly more prevalent in men, especially macrovascular diseases (10.8% vs. 7.8%, *p* < 0.001). As expected, microvascular complications, especially retinopathy, increased with diabetes duration and age.

HbA1c levels did not differ significantly according to the presence of any form of cardiovascular disease (Table S3 in Supplementary Materials).

Table 3 shows the current insulin therapy of patients with T1D stratified by age and also according to the absence/presence of cardiovascular disease. There were some differences in therapy between age groups. A greater percentage of people in the younger age groups were taking short and rapid acting insulins compared to those aged >40 years. Conversely, the use of pre-mixed insulins and NPH insulin was more prevalent in older people. Moreover, the use of pre-mixed and NPH insulins was more frequent in the presence of cardiovascular disease.

### 3.2. Control of Cardiovascular Risk-Factors

**Figure 2:**
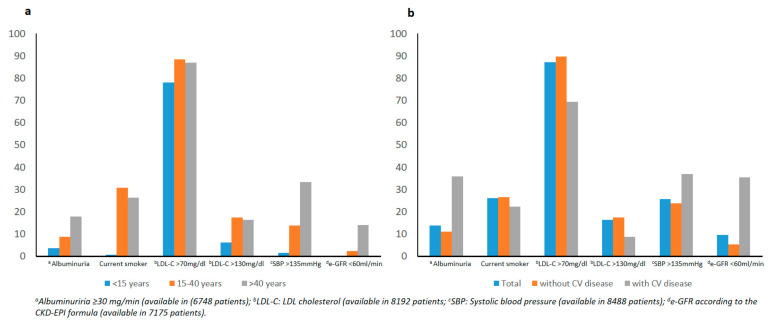
Prevalence of cardiovascular risk factors in T1D patients according to current age (**a**) and the absence/presence of cardiovascular disease (**b**).

Figure 2 shows the extent to which target values for different risk factors, other than HbA1c, were not met in this population of patients with T1D stratified according to current age (Figure 2a) and the presence of cardiovascular disease (Figure 2b). The proportion of patients with LDL-C below 70 mg/dl was only 12.8% (30.7% for patients with cardiovascular disease). The percentage of patients without cardiovascular disease on target for an LDL-C <130 mg/dl increased to 82.5%. It is noteworthy that 26.5% of the patients without and 22.4% with cardiovascular disease were current smokers. Moreover, the prevalence of SBP above 135 mmHg was 37% in patients with known cardiovascular disease.

## 4. Discussion

This study describes the clinical characteristics and the glycemic control and cardiovascular risk factor control in a large population-based cohort of 15,008 patients with T1D from Catalonia, a Spanish region in the Mediterranean area, using data extracted from a database not specifically designed for the study of diabetes. To our knowledge, this is the larger population-based study addressing this issue in southern Europe.

With some exceptions [19,20,21], the largest population-based studies on T1D are based on specifically dedicated registries [14,22,23], yielding somewhat conflicting results compared to data extracted from general healthcare databases [19]. We used an anonymous healthcare database that extracts data from multiple linked primary and secondary care sources, including the primary healthcare database that operates in most primary care centers in Catalonia. Even though T1D is a disease usually managed at the specialist level, the link to the primary health care database through drug and test strips claims allows its use in the study of the disease, with the advantage of recovering all those patients without regular contact at the specialist level, a potential pitfall of some specific databases [19]. After retrieving all potential cases through ICD-10 codes, we applied a restrictive selection strategy, excluding, basically, patients treated with additional antidiabetic drugs, in order to ensure the evaluation of patients with “true” T1D only. Although this strategy has most probably left some patients with T1D out of the study, the percentage of patients excluded was low and lower than patients treated with additional antidiabetic drugs in other studies [14].

The median HbA1c of the study population is in line with other studies [22,23], underscoring the difficulty of achieving optimal glycemic targets in these patients [24]. In this sense, only 20% of patients achieved an HbA1c < 7%. However, using less stringent targets, 51.7% of patients had an HbA1c level of <8% (64 mol/mol), which in some circumstances might be considered as an acceptable level of control [10]. Glucose control was totally superimposable in both sexes. Conversely, some differences were observed with increasing age, especially lower prevalence of very poor control (HbA1c >9% (75 mmol/mol)), a finding reported in most studies [14,19,20,22]. No clear differences in glucose control were observed regarding the duration of diabetes.

HbA1c concentrations did not differ according to gender. However, the prevalence of complications, especially macrovascular complications, was higher in males. Although some studies have reported that women with type 1 diabetes have a similar burden of cardiovascular disease to that of men [25], our results are in line with other findings that show that among type 1 diabetic individuals, females have a lower prevalence of cardiovascular disease when compared to males [17].

As expected, microvascular diabetic complications increased with diabetes duration and age, especially the prevalence of retinopathy, the most prevalent complication, and neuropathy. Interestingly, the prevalence of retinopathy was lower than in a similarly designed study from the UK [26]. Similarly, the prevalence of other microvascular complications was lower than previously published results [27]. Macrovascular complications showed a similar pattern that of microvascular complications but with much lower prevalence. In this case, the prevalence was similar to other studies except for peripheral vascular disease in which again the prevalence was lower than reported (14).

Insulin treatment patterns tended towards less complex regimens with increasing age and also in the presence of cardiovascular disease. This finding can be justified, in part, by the need for simpler treatment strategies in some circumstances and also by therapeutic inertia in patients used to and adapted to older treatment regimens. Although these simpler treatments are not commonly used nowadays, they have an acceptable efficacy and are not usually associated with an increased risk of hypoglycemia [28]. Unfortunately, the database does not include information about the use of insulin pumps, habits of glucose testing, or the use of continuous monitoring that could be useful in evaluating ways to improve glucose control [22].

Addressing the control of cardiovascular risk factors in patients with T1D in primary prevention is an issue with clear uncertainties since all current recommendations are based on intervention studies in type 2 diabetes [29]. In the present study, SBP was controlled at acceptable levels, but most patients were above the recommended LDL-C targets for patients at high risk for cardiovascular disease [30]. However, the degree of control of LDL-C is comparable to other studies [14,23,31]. On the other hand, the lack of adequate control of risk factors in patients with T1D and known cardiovascular disease is worrisome since the evidence that favors strict lipid and blood pressure control in this clinical setting is very robust [32,33]. The prevalence of smoking in this cohort was higher than might be expected, especially in the presence of cardiovascular disease. This finding is of concern, and more emphasis on smoking cessation and prevention programs may be warranted in the management of these patients. Of note, while our population compares poorly with some [23,34], some cohorts in other countries have also shown a similar or higher prevalence of smoking [14,17], suggesting the importance of smoking interventions elsewhere and in general. Finally, the prevalence of overweight and obesity was higher than previously reported [35], mirroring the increased prevalence of this emerging risk factor in the background population.

### Limitations

The present study has some limitations. The study relies on recorded codes without external validation measures. Although we have taken steps to ensure the validity of the diagnosis of T1D, the implementation of the clinical software on which the database was built began in 2001 and, therefore, we cannot exclude the possibility of the miscoding of some patients with T1D as type 2 diabetes [36], especially in the older population with a diagnosis before that date, possibly reducing the power of the study to evaluate a relevant proportion of patients with long-term T1D. The selected population also has some limitations, given that our objective was to give a reliable picture of the clinical situation of T1D patients, we decided to apply very restricting selection criteria, excluding patients with a T1D diagnosis but treated with glucose-lowering agents other than insulin. Moreover, the concomitant recording of T1D and T2D diagnosis, e.g., E10.9 plus E11.3, led to exclusion. This selection strategy gives us confidence that the patients studied have really T1D at the expense of excluding patients with T1D, and therefore the study cannot be seen as epidemiologic but rather as depicting the clinical state of patients with T1D in, probably, the best scenario possible. Furthermore, the possibility of underreporting of diabetes complications cannot be excluded. These are common limitations of current primary-care-based electronic record databases highlighting the need for additional validation studies using external databases, the development of internal control algorithms, and the comparison of the results to other similar studies. Another limitation is the proportion of missing clinical, i.e., blood pressure and BMI, and laboratory data for a significant proportion of the cohort. This fact may be due to the heterogeneity of providers of the public health system in Catalonia, which may have limited the transfer of some laboratory data to the database, although there is also a possibility that a clinical or laboratory assessment was not performed during the study year. Moreover, we did not have any information on the use of insulin pumps that may add important insights into metabolic control differences. Furthermore, with regard to acute complications, we only had information on recorded severe hypoglycemia, so the burden of symptomatic hypoglycemia or diabetic ketoacidosis could not be assessed. Finally, the cross-sectional design of the study precludes its use in morbidity and mortality prediction models.

## 5. Conclusions

We provide a reliable snapshot of the clinical situation of a large population of patients with T1D in Catalonia, which, in general, is similar to that reported in other western areas. The degree of metabolic control, although comparable to other studies, shows an important gap between the recommended targets and the reality of most patients, urging the need for new approaches in their clinical care. Of special importance is the finding of a lack of adequate control of cardiovascular risk factors in a significant proportion of patients with known cardiovascular disease. The current findings allow the identification of gaps in the clinical care of these patients and might lead to the implementation of strategies to improve it.

## Figures and Tables

**Table 1:** Clinical characteristics and treatments of the study population according to sex.

KERRYPNX	Total (*n* = 15,008)	Males (*n* = 8481)	Females (*n* = 6527)
Current age (years)	42 (31–53)	42 (31–52)	42 (30–54)
Age at diagnosis (years)	27.9 (15.5–39.2)	28.2 (16.5–38.8)	27.3 (14.1–39.8)
Diabetes duration (years)	11.8 (6.8–16.0)	11.7 (6.9–15.7)	11.8 (6.6–16.3)
<10 years	5814 (38.7)	3303 (38.9)	2511 (38.5)
10–19 years	6661 (44.4)	3798 (44.7)	2863 (43.9)
>19 years	2533 (16.9)	1380 (16.3)	1153 (17.7)
HbA1c (%) (*n* = 9085)	7.9 (7.1–8.8)	7.9 (7.1–8.9)	7.8 (7.1–8.8)
BMI (kg/m^2^) (*n* = 6610)	25.2 (22.4–28.5)	25.5 (22.8–28.3)	24.9 (21.9–28.7)
Overweight (BMI 25–29.9)	2342 (35.4)	1414 (39.3)	928 (30.8)
Obesity (BMI > 29.9)	1091 (16.5)	539 (15.0)	552 (18.3)
Smoking habit			
Current smoker	3921 (26.1)	2488 (29.3)	1433 (22.0)
Ex-smoker	3012 (20.1)	1910 (22.5)	1102 (16.9)
Hypertension			
*n* (%)	3459 (23.0)	2027 (23.9)	1432 (21.9)
Systolic BP (mmHg) (*n* = 8488)	128 (118–136)	130 (120–137)	124 (113–134)
Diastolic BP (mmHg) (*n* = 8488)	74 (67–80)	75 (68–80)	72 (66–79)
Hypercholesterolemia			
n (%)	3429 (22.8)	1959 (23.1)	1470 (22.5)
Total cholesterol (mg/dl) (*n* = 9287)	179 (157–203)	175 (153–199)	184 (162–207)
HDL-C (mg/dl) (*n* = 8787)	57 (48–69)	53 (44–63)	63 (53–75)
LDL-C (mg/dl) (*n* = 8192)	100 (82–120)	100 (81–120)	101 (83–120)
Triglycerides (mg/dl) (*n* = 8361)	83 (65–119)	88 (67–128)	79 (63–108)
GFR ^1^ (mL/min/1.72 m^2)^ (*n* = 7175)	90 (65–90)	90 (66–90)	90 (63.4–90)
Any antihypertensive medication *n* (%)	4076 (27.2)	2395 (28.2)	1681 (25.8)
ACEI/ARB *n* (%)	2816 (18.8)	1685 (19.8)	1136 (17.4)
Any lipid lowering agent *n* (%)	4563 (30.4)	2708 (31.9)	1855 (28.4)
Statins *n* (%)	4359 (29.0)	2565 (30.2)	1794 (27.5)
Aspirin *n* (%)	2339 (15.6)	1460 (17.2)	879 (13.5)

All comparisons were significant with a *p*-value < 0.005, except for HbA1c (*p* = 0.586), registered hypercholesterolemia (*p* = 0.37), LDL-cholesterol (*p* = 0.25), and BMI value (*p* = 0.17). Data are median (IQR) or *n* (%). Where indicated, the *n* value denotes the number of study patients with available data during the study period. ^1^ GFR according to the CKD-EPI formula. BMI: Body mass index, BP: Blood pressure, HDL-C: HDL-cholesterol, LDL-C: LDL-cholesterol, ACEI/ARB: Angiotensin-converting enzyme inhibitors/angiotensin II receptor blockers.

**Table 2:** Prevalence *n* (%) of diabetes-related micro- and macro-vascular complications, as assessed by ICD code records and laboratory data according to sex, diabetes duration, and current age.

	Total	Sex		Diabetes Duration (Years)			Current Age (Years)		
		Males	Females	<10	11–19	>19	<15	15–40	>40
*n*	15,008	8481	6527	5814	6661	2533	1116	5928	7964
Retinopathy	2982 (19.9)	1779 (21.0)	1203 (18.4)	559 (9.6)	1422 (21.3)	1001 (39.5)	2 (0.2)	622 (10.5)	2358 (29.6)
Albuminuria (mg/g creatinine) ^1^									
								
1	678	395	283	245	286	147	8	159	511
30–300	(10.0)	(10.5)	(9.4)	(10.0)	(9.5)	(11.5)	(3.7)	(6.2)	(12.9)
	258	169	89	80	114	64	0	66	192
>300	(3.8)	(4.5)	(3.0)	(3.3)	(3.8)	(5.0)	(0)	(2.6)	(4.9)
Impaired renal function ^2^	677 (9.4)	340 (8.7)	337 (10.4)	249 (9.9)	282 (8.4)	146 (11.1)	0 (0)	61 (2.3)	616 (13.9)
Dialysis	126 (0.8)	78 (0.9)	48 (0.7)	47 (0.8)	54 (0.8)	25 (1.3)	0 (0)	14 (0.2)	112 (1.4)
Neuropathy	1047 (7.0)	619 (7.3)	428 (6.6)	257 (4.4)	465 (7.0)	325 (12.8)	1 (0.1)	130 (2.2)	916 (11.5)
Any microvascular disease	3661 (24.4)	2132 (25.2)	1522 (23.3)	857 (14.7)	1700 (25.5)	1104 (43.6)	10 (0.9)	767 (12.9)	2884 (36.2)
Ischemic heart disease	669 (4.5)	425 (5.0)	244 (3.7)	209 (3.6)	267 (4.0)	193 (7.6)	1 (0.1)	27 (0.5)	641 (8.0)
Cerebral vascular disease	490 (3.3)	323 (3.8)	167 (2.6)	157 (2.7)	215 (3.2)	118 (4.7)	2 (0.2)	24 (0.4)	464 (5.8)
Peripheral vascular disease	610 (4.1)	440 (5.2)	170 (2.6)	198 (3.4)	253 (3.8)	159 (6.3)	0 (0)	26 (0.4)	584 (7.3)
Any macrovascular disease	1408 (9.4)	919 (10.8)	489 (7.5)	445 (7.7)	592 (8.9)	371 (14.6)	3 (0.3)	71 (1.2)	1334 (16.8)
Heart failure	319 (2.1)	184 (2.2)	135 (2.1)	125 (2.1)	125 (1.9)	69 (2.7)	0 (0)	5 (0.1)	314 (3.9)

All comparisons were significant with a *p*-value < 0.005 except for: Albuminuria according to diabetes duration (*p* = 0.018), dialysis according to gender (*p* = 0.241), and diabetes duration (*p* = 0.614), neuropathy according to gender (*p* = 0.081), and heart failure according to gender (*p* = 0.689) and diabetes duration (*p* = 0.034). ^1^ Available for 6748 patients (2999 females). ^2^ Defined as estimated glomerular filtration rate (e-GFR) according to the CKD-EPI formula <60 mL/min, available for 7170 patients (2903 females).

**Table 3:** Current insulin therapy for patients with T1D stratified by age group and presence of cardiovascular disease.

	Total	Current Age (Years)			Cardiovascular Disease ^1^	
		<15	15–40	>40	No	Yes
*n*	15,008	1116	5928	7964	13,506	1502
Insulin, short acting, *n* (%)	14,129 (94.1)	1096 (98.2)	5758 (97.1)	7275 (91.3)	12,867 (95.3)	1262 (84.0)
Insulin, pre-mixed, *n* (%)	1178 (11.9)	39 (3.5)	449 (7.6)	1292 (16.2)	1465 (10.8)	315 (20.9)
Insulin, NPH, *n* (%)	680 (4.5)	51 (4.6)	116 (2.0)	513 (6.4)	528 (3.9)	152 (10.1)
Insulin, long acting analogs, *n* (%)	12,719 (84.7)	990 (88.7)	5284 (89.1)	6445 (80.9)	11,572 (85.6)	1147 (76.3)

All comparisons were significant with a *p*-value < 0.001. ^1^ Cardiovascular disease defined as recorded diagnosis of ischemic heart disease, cerebral vascular disease.

## Data Availability

The data presented in this study are available on request from the corresponding author.

## Supplementary Materials

The following are available online at https://www.mdpi.com/article/10.3390/jcm10071536/s1, Table S1: ICD-10 codes used in the study, Figure S1: Flow diagram of the inclusion process, Figure S2: Distribution of age at diagnosis according to gender.
