# Supplementary material for: Clinical Characteristics and Degree of Glycemic and Cardiovascular Risk Factor Control in Patients with Type 1 Diabetes in Catalonia (Spain)
Source: J Clin Med. 2021 Apr 6;10(7):1536. doi: 10.3390/jcm10071536 (PMC8038775; doi:10.3390/jcm10071536)
Supplement: Supplementary file 1 [file jcm-10-01536-s001.pdf]

Supplementary

Table S1. ICD-10 codes used in the study.

ICD-10 CODE	Definition
**Inclusion criteria**	
E10	Type 1 diabetes mellitus
E10.0	Type 1 diabetes mellitus, with coma
E10.1	Type 1 diabetes mellitus, with ketoacidosis
E10.2	Type 1 diabetes mellitus, with renal complications
E10.3	Type 1 diabetes mellitus, with ophthalmic complications
E10.4	Type 1 diabetes mellitus, with neurological complications
E10.5	Type 1 diabetes mellitus, with peripheral circulatory complications
E10.6	Type 1 diabetes mellitus, with other specified complications
E10.7	Type 1 diabetes mellitus, with multiple complications
E10.8	Type 1 diabetes mellitus, with unspecified complications
E10.9	Type 1 diabetes mellitus, without complications
**Hypertension**	
I10	Essential (primary) hypertension
I11	Hypertensive heart disease
I11.0	Hypertensive heart disease with (congestive) heart failure
I11.9	Hypertensive heart disease without (congestive) heart failure
I12	Hypertensive renal disease
I12.0	Hypertensive renal disease with renal failure
I12.9	Hypertensive renal disease without renal failure
I13	Hypertensive heart and renal disease
I13.0	Hypertensive heart and renal disease with (congestive) heart failure
I13.1	Hypertensive heart and renal disease with renal failure
I13.2	Hypertensive heart and renal disease with both (congestive) heart failure and renal failure
I13.9	Hypertensive heart and renal disease, unspecified
**Hypercholesterolemia**	
E78.0	Pure hypercholesterolaemia
E78.1	Pure hyperglyceridaemia
E78.2	Mixed hyperlipidaemia
E78.3	Hyperchylomicronaemia
E78.4	Other hyperlipidaemia
E78.5	Hyperlipidaemia, unspecified
**Smoking**	
Z72.0	Tobacco use
**Acute diabetes complications**	
E10.0	Type 1 diabetes mellitus, with coma
E16.0	Drug-induced hypoglycaemia without coma
E16.2	Hypoglycaemia, unspecified
**Microvascular complications**	
E10.5	Type 1 diabetes mellitus, with peripheral circulatory complications
H36.0	Diabetic retinopathy (E10-E14 with common fourth character .3)
E10.2	Type 1 diabetes mellitus, with renal complications
Z99.2	Dependence on renal dialysis
E10.4	Type 1 diabetes mellitus, with neurological complications
G63.2	Diabetic polyneuropathy (E10-E14 with common fourth character .4)
**Macrovascular complications**	
E10.4	Type 1 diabetes mellitus, with neurological complications

I70.2	Atherosclerosis of arteries of extremities
I73	Other peripheral artery diseases
I73.8	Other specified peripheral vascular diseases
I73.9	Peripheral vascular disease, unspecified
I20	Angina pectoris
I21	Acute myocardial infarction
I22	Subsequent myocardial infarction
I23	Certain current complications following acute myocardial infarction
I24	Other acute ischaemic heart diseases
I25	Chronic ischaemic heart disease
I63	Cerebral infarction
I64	Stroke, not specified as haemorrhage or infarction
I67.2	Cerebral atherosclerosis
I67.3	Progressive vascular leukoencephalopathy
I67.4	Hypertensive encephalopathy
I67.8	Other specified cerebrovascular diseases
I67.9	Cerebrovascular disease, unspecified
I69	Sequelae of cerebrovascular disease
G45	Transient cerebral ischaemic attacks and related syndromes
G46	Vascular syndromes of brain in cerebrovascular diseases
**Heart failure**	
I150	Heart failure
I11.0	Hypertensive heart disease with (congestive) heart failure
I13.0	Hypertensive heart and renal disease with (congestive) heart failure
I13.2	Hypertensive heart and renal disease with both (congestive) heart failure and renal failure

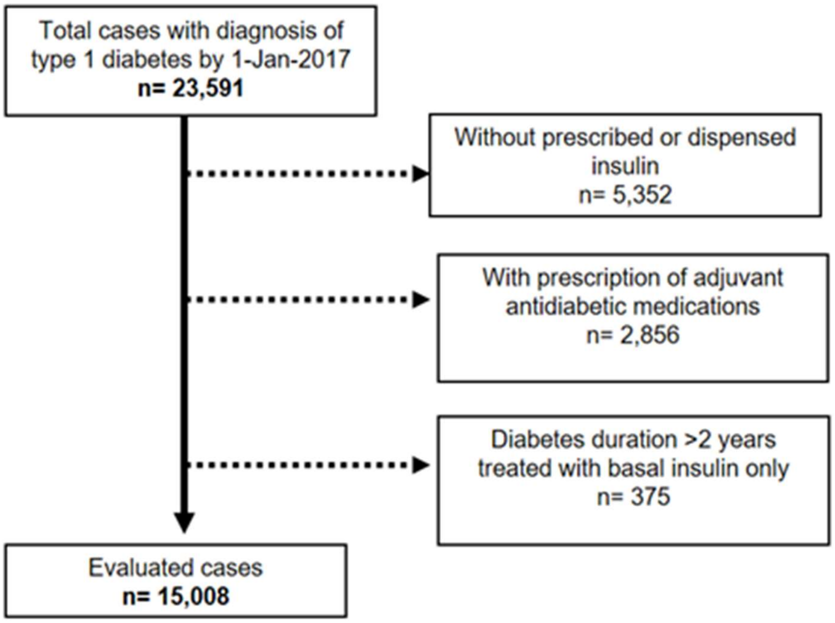

Figure S1. Flow diagram of the inclusion process.

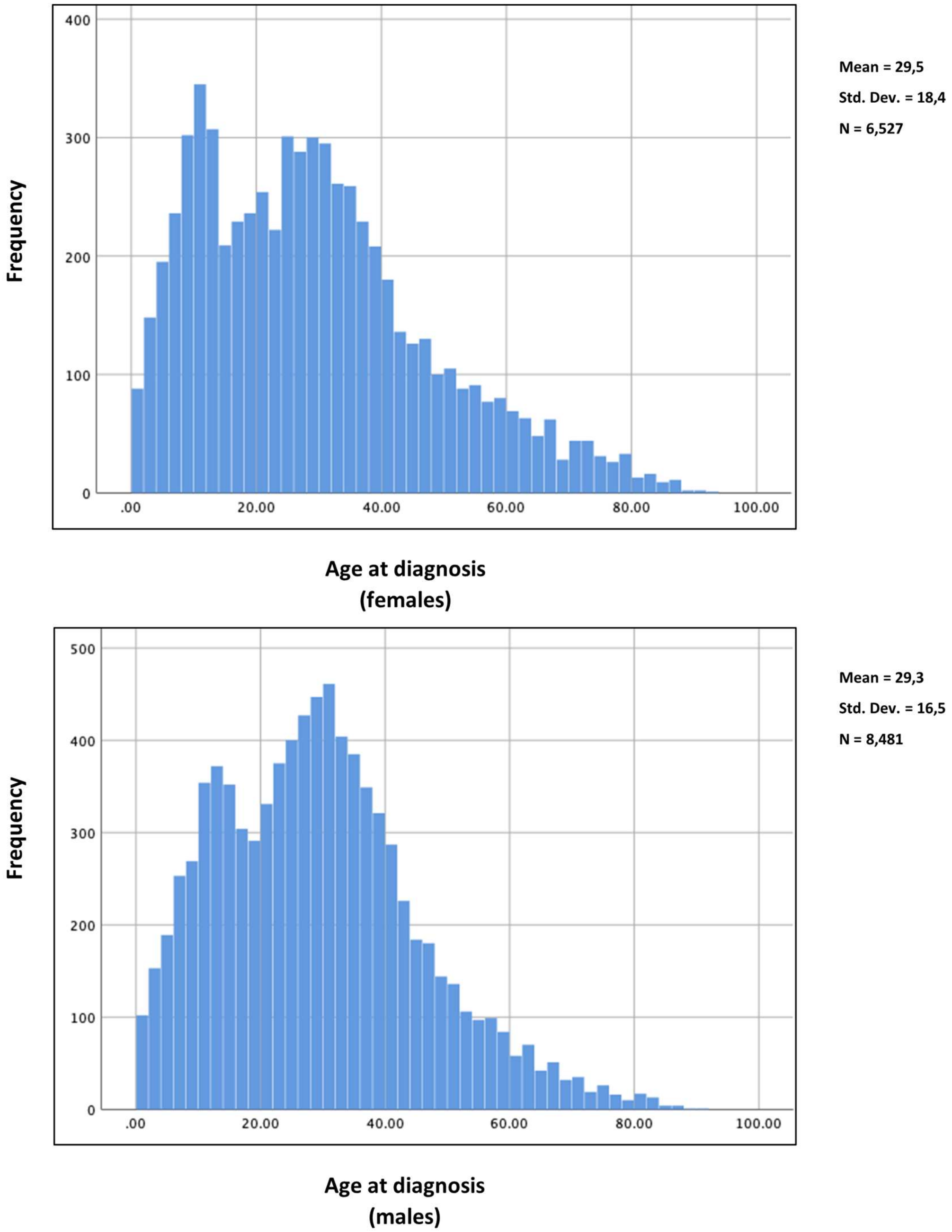

Figure S2. Distribution of age at diagnosis according to gender.

Table S2. Missing values of numerical variables n (%).

Parameter	Missing Values (%)
HbA1c	5505 (36.7%)
Blood pressure[1]	5884 (39.2%)
Total Cholesterol	5721 (38.1%)
HDL cholesterol	6221 (41.5%)
LDL Cholesterol	6816 (45.4%)
Triglycerides	6647 (44.3%)
BMI	8398 (56.0%)
GFR[2]	7833 (52.2%)
Albumine/creatinine ratio	8260 (55.0%)

[1] Systolic and diastolic blood pressure; [2] GFR according to the CKD-EPI formula.

Table S3. HbA1c levels according to the presence of cardiovascular disease and cardiovascular risk factors.

	**Mean HbA1c**	***p***
Coronary heart disease		
No	8.13 ± 1.51	0.113
Yes	8.02 ± 1.45	
Stroke		
No	8.12 ± 1.51	0.404
Yes	8.06 ± 1.32	
Peripheral artery diseasese		
No	8.13 ± 1.51	0.749
Yes	8.10 ± 1.51	
Hypertension		
No	8.17 ± 1.55	0.000
Yes	8.01 ± 1.38	
Hypecholesterolemia		
No	8.11 ± 1.53	0.085
Yes	8.17 ± 1.44	
